# Manual Acupuncture for Optic Atrophy: A Systematic Review and Meta-Analysis

**DOI:** 10.1155/2019/1735967

**Published:** 2019-01-01

**Authors:** Fang-Yuan Zhi, Jie Liu, Xiao-Peng Ma, Jue Hong, Ji Zhang, Dan Zhang, Yue Zhao, Li-Jie Wu, Yan-Ting Yang, Dan-Yan Wu, Chen Xie, Ling-Xiang Wu, Cui-Hong Zhang

**Affiliations:** ^1^Shanghai University of Traditional Chinese Medicine, Shanghai 201210, China; ^2^Shanghai Research Institute of Acupuncture and Meridian, Shanghai 200030, China

## Abstract

*Objectives. *This systematic review aims to critically evaluate the efficacy of manual acupuncture for optic atrophy. Eight English and Chinese databases, including Cochrane Library, EMbase, PubMed, Chinese National Knowledge Infrastructure (CNKI), Wanfang Database, China Science and Technology Journal Database (VIP), and Chinese Biomedical Literature Database (CBM), as well as ongoing trials registered with the WHO International Clinical Trials Registry Platform, were searched to identify eligible randomized controlled trials (RCTs) studying manual acupuncture for optic atrophy compared to medication alone. The quality of evidence was assessed using Cochrane Collaboration's risk of bias tool. Meta-analysis was performed using Review Manager version 5.3. Nine studies were identified and included for meta-analysis. The meta-analysis showed significant differences in favor of manual acupuncture or manual acupuncture plus medication compared with medication alone in the following outcome measures: visual acuity (MD = 0.18, 95% CI [0.17, 0.20],* P* < 0.00001), mean sensitivity of visual field (MD = 2.11, 95% CI [1.90, 2.32],* P *< 0.00001), the latent period of P-VEP100 (MD = -6.80, 95% CI [-8.94, -4.66],* P* < 0.00001), the total effectiveness (264 eyes) (OR = 3.22, 95% CI [1.88, 5.51],* P*<0.0001), and the total effectiveness (344 participants) (OR = 4.29, 95% CI [2.56, 7.19],* P* < 0.00001). Despite statistical advantages of manual acupuncture in the literature, due to serious methodological flaws in study design, it cannot be concluded that manual acupuncture is more effective than medicine alone. It is essential that a properly controlled clinical trial is designed and controls are established to exclude placebo effects.

## 1. Introduction

Optic atrophy is a condition in which the retinal ganglion cells and their axons degenerate. It usually manifests as variable degrees of visual dysfunction and a gray or pale optic disc [1–3]. Optic atrophy can be an independent disease but meanwhile the endpoint of multiple eye or somatic diseases [4, 5]. It has many risking factors, such as infection, ischemia, compression, trauma, toxic, degeneration, demyelination, and genetic diseases [6–9]. Epidemiological surveys have showed that optic atrophy ranks the second among visual dysfunctions in children [10, 11]. Due to its complex causes, high incidence, long extension, high possibility causing blindness, and significant influence on life and work, optic atrophy has drawn great attention in the medical field [12, 13].

Optic nerve is made of axons which do not regenerate once damaged [14, 15]. Among all the choices provided, nutraceuticals, vasodilators, improving circulation, peripheral nerve transplantation, umbilical cord blood mesenchymal stem cell, and gene therapy, as well as the integrative treatment combining traditional Chinese herbal medications, there is no reliable cure yet for optic atrophy [16–23].

Acupuncture therapy has a long history of treating optic atrophy and is well-accepted by patients for its convenience and therapeutic benefit [24]. Studies show that acupuncture can activate the bioelectricity in visual center, improve optic nerve cell metabolism, and promote the local blood circulation, which allows the partially damaged optic nerves to be repaired [25, 26]. Acupuncture at Feng-chi (GB20) can regulate the tension and elasticity of blood vessels of brain, thus helping the blood circulation of brain [27] and also the vision via improving ocular blood circulation [28]. Acupuncture at Tai-chong (LR3) can shorten the latency of pattern visual evoked potential (P-VEP) P100 and improve optic nerve conduction [29]. Some studies hold that the heat sensation produced via acupuncture at an acupoint can enhance blood circulation, improve microcirculation and nourish optic nerves [30]. The above studies have provided scientific evidence for the treatment of optic atrophy with acupuncture. To reach a more reliable conclusion, we carried out this systematic review and meta-analysis to evaluate the efficacy of manual acupuncture for optic atrophy by collecting the eligible randomized clinical trials (RCTs).

## 2. Methods

### 2.1. Study Selection

Articles that meet the following criteria were included: (1) the included trials were randomized controlled trials studying manual acupuncture for treating optic atrophy; (2) the included patients were diagnosed with optic atrophy, regardless of the nationality, race, sex, age or causes (glaucoma, trauma, retinitis pigmentosa, age-related macular degeneration, optic nerve ischemia, inflammation, unknown causes, etc.); (3) we included studies on manual acupuncture or manual acupuncture with medication compared to medication alone. Here manual acupuncture referred to needles inserted to acupoints, excluding moxibustion, electroacupuncture, auricular acupoint therapy, bloodletting cupping, acupoint injection, acupoint sticking, acupoint thread embedding, etc.; (4) the primary outcome measures were as follows: visual acuity (VA), visual field (VF), pattern visual evoked potential (P-VEP), and total effectiveness (TE); (5) full text should be available.

Studies with the following situations were excluded: (1) acupuncture combined with other treatments including electrical stimulation, moxibustion, or Chinese herbal medicine; (2) the patients in control group not treated with medication; (3) studies that included other treatments in acupuncture group or control group; (4) full text should be available.

### 2.2. Search Strategy

Two authors worked independently on data retrieval, study selection, data extraction, and quality assessment to avoid incomplete search or data missing and ensure the objectivity and reasonability. The discrepancies between the two authors were solved by a third author. Eight databases were searched from inception to March 27, 2018, including Cochrane Library, EMbase, PubMed, Chinese National Knowledge Infrastructure (CNKI), Wanfang Database, China Science and Technology Journal Database (VIP), and Chinese Biomedical Literature Database (CBM), as well as ongoing clinical trials registered with the WHO International Clinical Trials Registry Platform. Search terms including “acupuncture”, “acupuncture therapy”, “needling”, “manual acupuncture”, “optic atrophy”, “optic neuropathy” and “clinical trial” were used independently or in combination for full-text retrieval. For example, the search terms used in PubMed were (“acupuncture” [MeSH Terms] OR “acupuncture” [All Fields] OR “acupuncture therapy” [MeSH Terms] OR (“acupuncture” [All Fields] AND “therapy” [All Fields]) OR “acupuncture therapy” [All Fields]) AND (“medicine” [MeSH Terms] OR “medication” [All Fields]OR drug[All Fields]) AND (“optic atrophy” [MeSH Terms] OR (“optic” [All Fields] AND “atrophy” [All Fields]) OR “optic atrophy” [All Fields] OR “optic neuropathy” [All Fields]) AND (randomized controlled trial [pt] OR controlled clinical trial [pt] OR randomized [tiab] OR clinical trials as topic [mesh: noexp] OR randomly [tiab] OR trial [ti]) NOT (animals [mh] NOT humans [mh]).

### 2.3. Data Extraction

The extracted information included author, title, publication year, study design, baseline, randomization method, allocation concealment, blinding method, follow-up, dropout and withdrawal, relapse, interventions, treatment duration, diagnostic criteria, inclusion criteria, exclusion criteria, efficacy evaluation standard, effective case number, total case number, outcome measurement indexes, and adverse events.

### 2.4. Quality Assessment

Two authors independently assessed the methodological quality using Cochrane Collaboration's risk of bias tool (Handbook 5.1) [31]. The assessed characteristics included random sequence generation; allocation concealment; blinding of participants, blinding of outcome assessors; incomplete outcome data; selective reporting; other bias. The above domains were evaluated and categorized into low risk, high risk, or unclear.

### 2.5. Statistical Analysis

The data were analyzed using Review Manager version 5.3 (Cochrane, London, UK). We examined heterogeneity among the studies using Chi-square test and Higgins *I*^2^ test. A fixed effects model was used when the heterogeneity was not significant, while a random effects model was adopted when the heterogeneity was significant. Meanwhile, subgroup analysis or sensitivity analysis would be performed to test the impact of the quality of the included trials. Odds ratio (OR) was used for dichotomous data and mean difference (MD) was used for continuous variables. The outcomes were expressed with 95%CI, and* P*<0.05 was indicative of significant difference between the experiment and control groups. For the continuous variables of VA, VF, and P-VEP, intragroup differences before and after treatment were used. When intragroup differences were not available, and the original report only gave the mean and standard deviation of the values before and after the treatment, the following formula would be applied:

(1) MeanE, change=MeanE, final−MeanE, baselineSDE change=SDE baseline2+SDE final2−2×Corr×SDE baseline×SDE final

Corr was set at 0.5.

## 3. Results

### 3.1. General Description of Literatures

**Figure 1:**
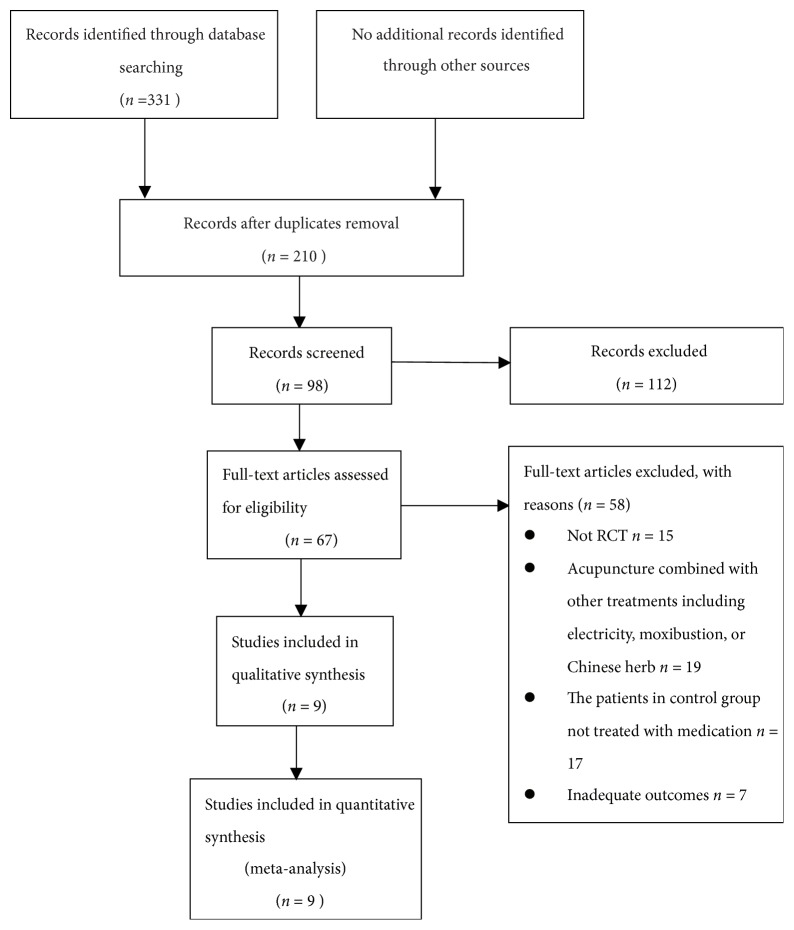
Flow chart of the study screening and selection process.

A total of 331 papers were identified at the initial search, including 327 papers written in Chinese and 4 in English. After removal of duplicates, review of titles, abstracts, and full texts, nine studies were finally included, all with manual acupuncture as the experiment intervention and medication alone as the control intervention (Figure 1). The nine papers were all in Chinese.

### 3.2. Study Characteristics

Table 1 describes the characteristics of the included studies. All of the studies were conducted in China and published in Chinese with a total of 513 participants: 262 in experiment groups and 251 in control groups. Of the nine included trials, three studies with manual acupuncture as the experiment groups and medication alone as the control groups; six studies with manual acupuncture plus medication and medication alone as the control groups (Table 1). The acupuncture characteristics of each study included in this meta-analysis are described in Table 2.

### 3.3. Quality of the Included Studies

**Figure 2:**
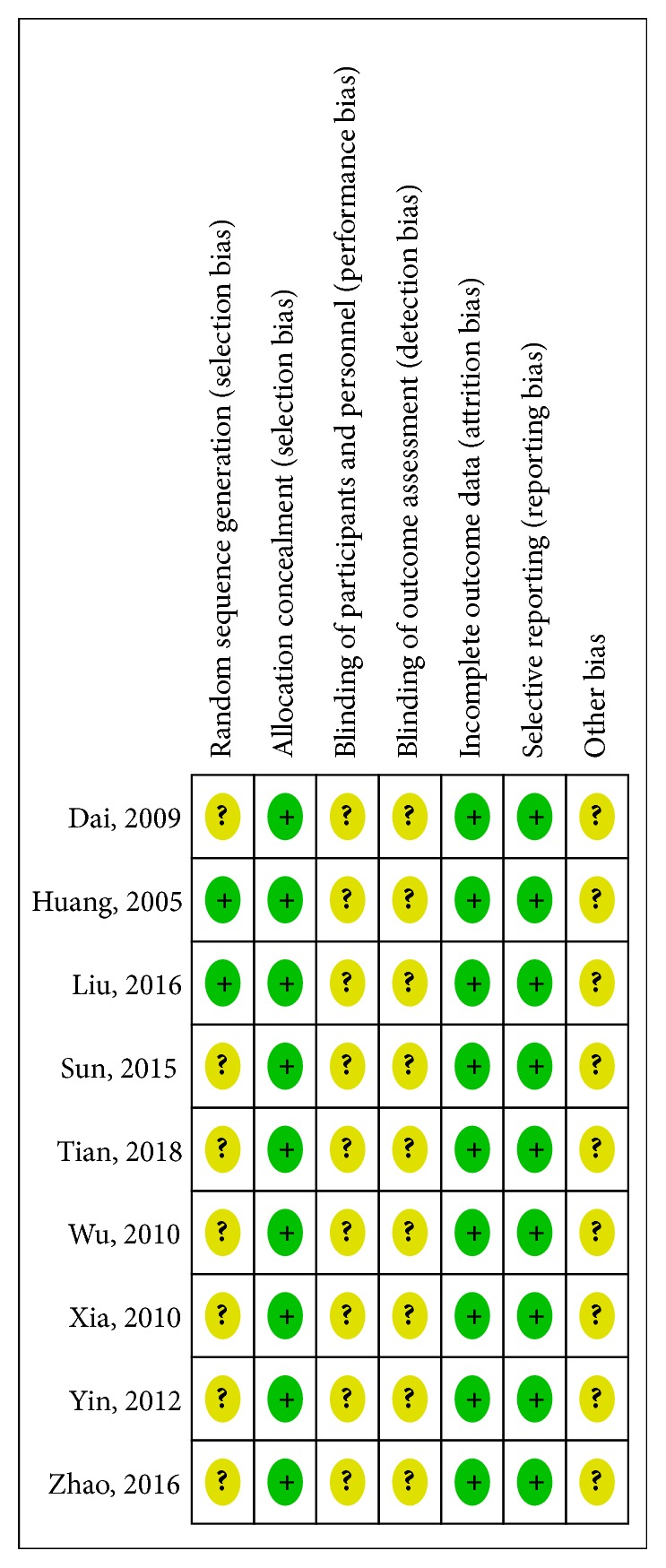
Risk of bias in the included studies: review authors' judgements about each risk of bias item for each included study. Note: “+”: low risk; “?”: unclear risk; “-”: high risk.

The included nine studies were all randomized controlled trials. Among which, only two studies [32, 40] described the random method. Liu 2016 [32] reported the use of random number table; Huang 2005 [40] used computer for randomization. The rest trials failed to describe which specific random method was used. All the studies recruited subjects based on inclusion and exclusion criteria and therefore the selection of patients can be considered to be low risk in selective bias. Baseline data were described in all studies and baseline comparability was claimed. No study mentioned the use of blinding. Dropout and loss to follow-up were not mentioned in any of the studies. But, based on the consistency of the study data, we still believe that the outcome data were complete. All the studies reported the complete outcome data. The sources of other bias in all studies were unclear. No study reported adverse events. (Figure 2)

### 3.4. Improvement of VA

**Figure 3:**
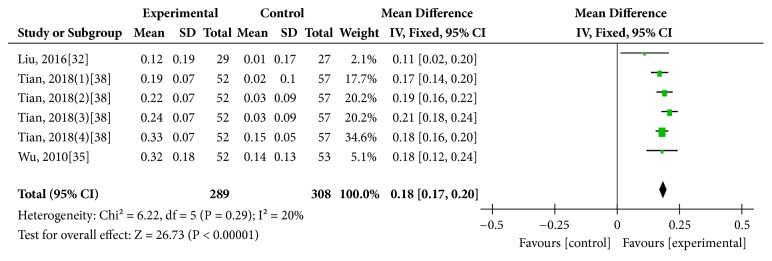
Comparison of VA between manual acupuncture and medication alone for optic atrophy.

Three studies [32, 35, 38] took the test of vision as one of the outcome measures. There were 289 eyes in the treatment group (including the number of eyes counted repeatedly) and 308 eyes in the control group (including the number of eyes counted repeatedly). The meta-analysis did not have significant heterogeneity (*I*^2^ = 20%,* P *= 0.29), so that a fix effects model was used. Regarding the improvement of vision, the result identified a significant difference favoring manual acupuncture compared with medication (MD = 0.18, 95% CI [0.17, 0.20],* P *< 0.00001). (Figure 3) Zhao, 2016 [33] only counted the number of the included patients, but failed to count the number of the affected eyes, so this study was not included in meta-analysis. However, its results also showed that manual acupuncture was better in improving the average visual acuity in optic atrophy compared with medication alone. The results of the above studies indicated that, in comparison with improving the average visual acuity, manual acupuncture was superior to medication alone.

### 3.5. Improvement of VF

**Figure 4:**
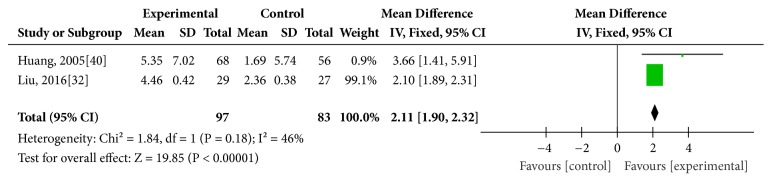
Comparison of mean sensitivity of VF between manual acupuncture and medication alone for optic atrophy.

Two studies [32, 40] reported mean sensitivity of VF in the outcome. There were 89 eyes in the experimental group and 75 eyes in the control. The meta-analysis showed no significant heterogeneity (*I*^2^ = 46%,* P *= 0.18), so that a fix effects model was used. The result showed that manual acupuncture was more effective than medication alone in improving mean sensitivity of visual field (MD = 2.11, 95% CI [1.90, 2.32],* P *< 0.00001). (Figure 4)

One trial [40] studied the average visual field defect and the result suggested that manual acupuncture could more significantly help to ameliorate the average visual field defect compared with medication alone. Another study [35] referred to the gray scale of visual field and reported that manual acupuncture was more effective in reducing the gray scale compared with medication alone. Nevertheless, only one trial was not sufficient to provide strong evidence to prove which treatment was more effective in terms of visual field defect and visual field gray scale.

### 3.6. Improvement of P-VEP

**Figure 5:**
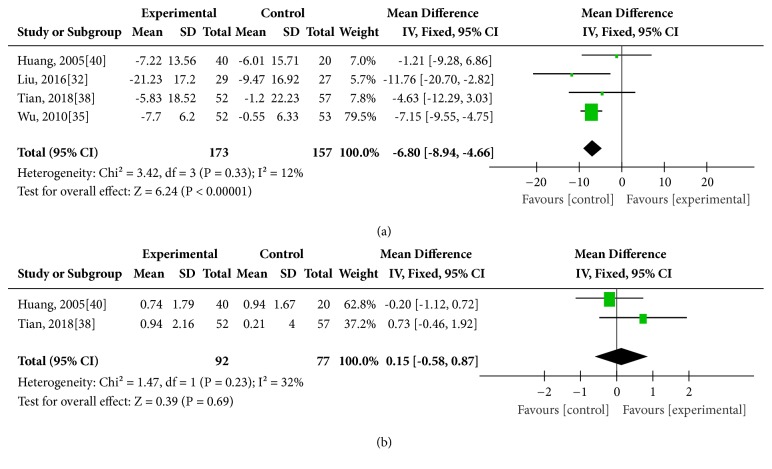
(a) Comparison of latency of wave P100 in P-VEP between manual acupuncture and medication alone for optic atrophy. (b) Comparison of amplitude of wave P100 in P-VEP between manual acupuncture and medication alone for optic atrophy.

Four studies [32, 35, 38, 40] took the latent period of P-VEP100 as one of the outcome measures. There were 173 eyes in the experimental group and 157 eyes in the control. The meta-analysis did not have significant heterogeneity (*I*^2^ = 12%,* P* = 0.33), so that a fix effects model was used. The result showed that manual acupuncture was more effective than medication alone in improving the latent period of P-VEP100 (MD = -6.80, 95% CI [-8.94, -4.66],* P* <0.0001). (Figure 5(a)) Sun, 2015 [34] only mentioned the latency after treatment, but did not provide the pretreatment level, neither the number of the affected eyes. As a result, this trial was not included in meta-analysis.

Two studies [38, 40] took amplitude of P-VEP100 as one of the outcome measures. There were 184 eyes in the experimental group and 77 eyes in the control group. The meta-analysis showed insignificant difference between manual acupuncture and medication alone (MD = 0.15, 95% CI [-0.58, 0.87],* P* = 0.69). Sun, 2015 [34] only mentioned the amplitude after the treatment, but it did not mention the pretreatment level or the number of eyes. Therefore, it was not included in meta-analysis. The results of the above studies showed that manual acupuncture had no significant advantage in improving the amplitude of the visual evoked potential P100 compared with medication alone. (Figure 5(b))

### 3.7. TE

**Figure 6:**
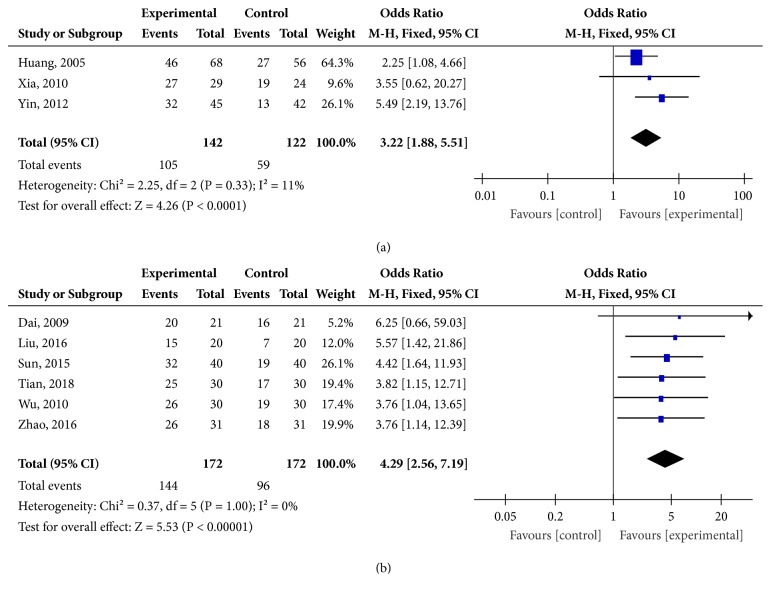
(a) Comparison of the total effectiveness between manual acupuncture and medication alone for optic atrophy (264 eyes). (b) Comparison of the total effectiveness between manual acupuncture and medication alone for optic atrophy (344 participants).

Regarding the total effectiveness, three studies [36, 39, 40] counted the number of the affected eyes: 142 eyes in the experimental group and 122 eyes in the control group. The meta-analysis did not have significant heterogeneity (*I*^2^ = 11%,* P* = 0.33), so that a fix effects model was used. The result showed that manual acupuncture was more effective than medication alone in improving total effectiveness (OR = 3.22, 95%CI [1.88, 5.51],* P *< 0.0001). (Figure 6(a))

In addition, six studies [32–35, 37, 38] counted the number of patients: 172 patients in the experimental group and 172 patients in the control group. The meta-analysis did not have significant heterogeneity (I^2^ = 0%,* P* = 1.00), so that a fix effects model was used. The result showed that manual acupuncture was more effective than medication alone in improving total effectiveness (OR = 4.29, 95%CI [2.56, 7.19],* P* < 0.00001). (Figure 6(b))

The results of the above studies indicated that manual acupuncture was superior to medication alone in improving the total effectiveness.

### 3.8. Sensitivity Analysis

Sensitivity analysis was performed by transforming the model of the effect. Sensitivity analysis revealed that each group has little difference after exchanging models. This indicates that the sensitivity of each group of data is low, that is, the small sample study has little effect on the combined effect. It shows that the stability of meta-analysis is higher (Table 3).

## 4. Discussion

Optic atrophy can be an independent disease but meanwhile the endpoint of a variety of diseases (glaucoma, retinitis pigmentosa, optic nerve ischemia, etc.), greatly affecting patient's quality of life. The causing factors are rather complicate and the prognosis is usually poor. So far, there is still no specific treatment for this condition. Therefore, it has become a difficult urgency to seek an effective treatment for optic atrophy. Acupuncture has demonstrated great advantage in treatment of optic diseases during the recent years, e.g., dry eye disease, amblyopia and glaucoma [41–43]. One study [44] showed that acupuncture might improve the visual function and the conductivity of optic nerves of the affected eye via evoking the remaining nerve fibers as long as the visual structure was not completely damaged. This suggests that acupuncture have certain improving effect on optic atrophy. In recent years, there have occurred more and more clinical studies on acupuncture-moxibustion treatment of optic atrophy. In order to understand whether manual acupuncture has reliable curative effect for optic atrophy, this study conducted a systematic review and meta-analysis by recruiting clinical papers studying manual acupuncture treatment of optic atrophy despite the reasons. The purpose was also to provide a reference for clinical practice.

Dai YL et al. [45] and Liu ML et al. [46] also performed meta-analysis of RCTs studying acupuncture for optic atrophy and held that acupuncture could produce satisfactory efficacy for this disease. However, they did not give a precise definition to the intervention of the included trials; as a result, trials using single and integrative acupuncture therapy were all covered, which inevitably caused a significant heterogeneity. To prevent this problem, in this study we limited the experiment intervention to manual acupuncture or manual acupuncture plus medication and the control intervention to medication alone, to more rigorously reflect the efficacy of acupuncture in treatment of optic atrophy.

Nine RCTs were finally included after rigorous design and screening. Among them, three studies [32, 35, 38] were included in meta-analysis for the comparison of VA improvement; two studies [32, 40] were included in meta-analysis for the mean sensitivity of VF; for the latency of P100, 4 studies [32, 35, 38, 40] were included in meta-analysis; for the comparison of P100 amplitude, 2 studies [38, 40] were included; for the effectiveness, three studies [36, 39, 40] counted the number of eyes, and six studies [32–35, 37, 38] counted the number of patients, and they were taken into meta-analysis, respectively. There was one study for visual field average defect [40] and visual field gray scale [35], so these two were not subjected to meta-analysis. Statistically speaking, our meta-analysis showed that manual acupuncture or manual acupuncture plus medication may be more effective than medication alone in the improvement of VA, mean sensitivity of VF, latency of wave P100 in P-VEP and the total effectiveness. The sensitivity analysis also proved the stability of the results. The evidences were insufficient to prove the advantage of manual acupuncture compared with medication alone in terms of visual field defect, visual field gray scale, and amplitude of P-VEP P100.

However, the following shortcomings may weaken the above conclusion: (1) only two studies clearly described the random method, while the rest only mentioned “randomization” without specific details, suggesting that there may be selective bias; (2) all the studies did not mention about blinding of participants or personnel, which may cause performance bias; (3) the publication language of all the included studies was Chinese, suggesting a possibility of publication bias; (4) none of the included studies reported follow-up and adverse reactions, suggesting that there may be other sources of bias.

The nine included RCTs generally had problems with sample size and methodological quality, so it is too early to obtain a valid conclusion. No serious adverse reactions, mild and occasional side effects, these merits allow acupuncture-moxibustion to be easily accepted. Although none of the nine studies reported adverse events, acupuncture at the intraorbital acupoints such as Jing-ming (BL2) and Qiu-hou (EX-HN7) may cause topical subcutaneous hemorrhage and hematoma. That is why patients are usually asked to press these areas for 2-3 min at the removal of needles.

In view of the problems existing in current clinical research, it is necessary to conduct a rigorous RCT on acupuncture treatment of optic atrophy in future studies. In the design and report of the trial, each step should be completed by strictly following the CONSORT [47] and STRICTA [48] statements, so as to standardize the research report and improve the quality. Future investigations should improve its methodological quality from the following aspects: (1) clinical studies need to be conducted in a more rigorous way and the researchers should elevate their comprehension of RCTs; (2) placebo such as sham acupuncture should be properly used as a control to rule out the placebo effect of acupuncture; (3) random methods, allocation concealment and implementation of blinding should be stressed to exclude investigator bias; (4) accurately describe the specific information about acupuncture method adopted in the trial, including main acupoints, insertion depth, duration (min), frequency, total session and needle type; (5) to conduct multicentered clinical trials, better with a large sample size and sufficient follow-up duration; (6) the evaluation system for optic atrophy should be standardized; (7) the recurrence and adverse events should be reported to further estimate the effectiveness and safety of manual acupuncture in the treatment of optic atrophy.

## 5. Conclusion

Despite statistical advantages of manual acupuncture in the literature, due to serious methodological flaws in study design, it cannot be concluded that manual acupuncture or manual acupuncture plus medication is more effective than medication alone. It is essential that a properly controlled clinical trial is designed and placebo effects are excluded.

## Figures and Tables

**Table 1:** Characteristics of the included studies.

**Study ID**	***n* (number of eye)**		**Gender (male/female)**		**Mean age (years)**		**Interventions**		**Outcomes**	**Study type**
	**E**	**C**	**E**	**C**	**E**	**C**	**E**	**C**		
Liu, 2016[32]	20(29)	20(27)	13/7	12/8	52±16	50±16	MA+M	M	VA, VF, P-VEP, TE	RCT
Zhao, 2016 [33]	31(NM)	31(NM)	20/11	16/15	50.22±12.30	19.59±11.08	MA+M	M	VA, TE	RCT
Sun, 2015 [34]	40(NM)	40(NM)	21/19	22/18	46.1±2.3	46.1±3.0	MA+M	M	P-VEP, TE	RCT
Wu, 2010 [35]	30(52)	30(53)	21/9	20/10	46.4±14.14	48.83±13.52	MA+M	M	VA, VF, P-VEP, TE	RCT
Xia, 2010 [36]	29(29)	24(24)	15/14	16/8	53.8±12.3	52.5±11.9	MA+M	M	TE	RCT
Dai, 2009 [37]	21(25)	21(25)	11/10	11/10	40.5	40.5	MA+M	M	TE	RCT
Tian, 2018 [38]	30(52)	30(57)	18/12	15/15	49.11±2.41	49.11±2.41	MA	M	VA, VF, P-VEP, TE	RCT
Yin, 2012 [39]	25(50)	25(50)	10/15	11/14	57.6	55.9	MA	M	TE	RCT
Huang, 2005 [40]	36(68)	30(56)	15/21	12/18	45.2	45.2	MA	M	VF, P-VEP, TE	RCT

Notes: E: experiment group; C: control group; MA: manual acupuncture; M: medication; VA: visual acuity; VF: visual field; P-VEP: pattern visual evoked potential; TE: total effectiveness; NM: not mentioned.

**Table 2:** Acupuncture details of the included studies.

**Study ID**	**Main acupoints**	**Insertion depths ** **(individual cun)**	**Duration (min)**	**Frequency**	**Total sessions**	**Needle type**	**Acupuncture rational**
Liu, 2016 [32]	Xin-ming I, Xing-ming II	1~1.5	3	once a day	30	0.30 mm × 40 mm	TCM

Zhao, 2016 [33]	Cheng-qi (ST1), Cuan-zhu (BL2), Yang-bai (GB14), Feng-chi (GB20)	0.5	20	once a day	30	NM	TCM

Sun, 2015 [34]	Qiu-hou (EX-HN7), Jing-ming (BL1), Feng-chi (GB20)	1~1.5	20	once a day	28	NM	TCM

Wu, 2010 [35]	Qiu-hou (EX-HN7), Jing-ming (BL1), Feng-chi (GB20)	1~1.5	20	once a day	28	NM	TCM

Xia, 2010 [36]	Cheng-qi (ST1), Tai-yang (EX-HN5), Cuan-zhu (BL2), Feng-chi (GB20)	0.5~1	30~45	once a day	42	NM	TCM

Dai, 2009 [37]	Jing-ming (BL1), Qiu-hou (EX-HN7), Cuan-zhu (BL2), Yang-bai (GB14), Cheng-qi (ST1), He-gu (LI4)	1~1.5	30	NM	60	NM	TCM

Tian, 2018[38]	Feng-chi (GB20), Tai-yang (EX-HN5), Tong-zi-liao(GB1), Si-zhu-kong(SJ23)	NM	3	NM	28	0.25 mm × 40 mm	TCM

Yin, 2012 [39]	Cheng-qi (ST1), Jing-ming (BL1)	1.2 ~ 1.3	30	NM	NM	0.30 mm × 40 mm	TCM

Hang, 2005 [40]	Bai-hui (DU20), Feng-chi (GB20), Qiu-hou (EX-HN7),Tai-yang (EX-HN5), Jing-ming (BL1), Cheng-qi(ST1)	NM	30	once a day	28d	0.35 mm × 40 mm	TCM

**Table 3:** Sensitivity analysis.

Outcomes	Effect Model	Effect Size (95% CI)	Z	P
Visual acuity	Fixed	0.18 (0.17, 0.20)	26.73	<0.00001
	Random	0.18 (0.17, 0.20)	22.86	<0.00001

Visual field mean sensitivity	Fixed	2.11 (1.90, 2.32)	19.85	<0.00001
	Random	2.46 (1.17, 3.75)	3.74	0.0002

Latency of wave P100 in P-VEP	Fixed	-6.80 (-8.94, -4.66)	6.24	<0.00001
	Random	-6.62 (-9.38, -3.86)	4.70	<0.00001

Amplitude of wave P100 in P-VEP	Fixed	0.15 (-0.58, 0.87)	0. 39	0.69
	Random	0.18 (-0.71, 1.08)	0.40	0.69

Total effectiveness (264 eyes)	Fixed	3.22 (1.88, 5.51)	4.26	<0.0001
	Random	3.26 (1.80, 5.89)	3.91	<0.0001

Total effectiveness (344 participants)	Fixed	4.29 (2.56, 7.19)	5.53	<0.00001
	Random	4.28 (2.55, 7.18)	5.52	<0.00001

## References

[1] Lenaers G., Hamel C. P., Delettre C. (2012). Dominant optic atrophy. *Orphanet Journal of Rare Diseases*.

[2] Kanamori A., Nakamura M., Yamada Y., Negi A. (2013). Spectral-domain optical coherence tomography detects optic atrophy due to optic tract syndrome. *Graefe's Archive for Clinical and Experimental Ophthalmology*.

[3] Zhang L., Shi W., Song L. (2015). A recurrent deletion mutation in OPA1 causes autosomal dominant optic atrophy in a Chinese family. *Scientific Reports*.

[4] Formichi P., Radi E., Giorgi E. (2015). Analysis of opa1 isoforms expression and apoptosis regulation in autosomal dominant optic atrophy (ADOA) patients with mutations in the opa1 gene. *Journal of the Neurological Sciences*.

[5] Osaguona V. B., Okeigbemen V. W. (2015). Nonglaucomatous optic atrophy in Benin City. *Annals of African Medicine*.

[6] Espino Barros Palau A., Morgan M. L., Lee A. G. (2014). Bilateral optic atrophy in endemic typhus. *Canadian Journal of Ophthalmology*.

[7] Heidary G., Calderwood L., Cox G. F. (2014). Optic atrophy and a leigh-like syndrome due to mutations in the C12orf65 gene: Report of a novel mutation and review of the literature. *Journal of Neuro-Ophthalmology*.

[8] Kumar N., Singh A., Saxena R., Menon V. (2014). An unusual cause of optic atrophy in a child. *Indian Journal of Ophthalmology*.

[9] Işcan Y., Coskun Ç., Öner V., Türkçü F. M., Tas M., Alakus M. F. (2013). Bilateral total optic atrophy due to transdermal methanol intoxication. *Middle East African Journal of Ophthalmology*.

[10] Vaphiades M. S., Brodsky M. C. (2012). Pediatric Optic Atrophy. *International Ophthalmology Clinics*.

[11] Chinta S., Wallang B. S., Sachdeva V., Gupta A., Patil-Chhablani P., Kekunnaya R. (2014). Etiology and clinical profile of childhood optic nerve atrophy at a tertiary eye care center in South India. *Indian Journal of Ophthalmology*.

[12] Zheng L., Do H. H.-J., Sandercoe T., Jamieson R. V., Grigg J. R. (2016). Changing patterns in paediatric optic atrophy aetiology: 1979 to 2015. *Clinical & Experimental Ophthalmology*.

[13] Chun B. Y., Rizzo J. F. (2016). Dominant optic atrophy: Updates on the pathophysiology and clinical manifestations of the optic atrophy 1 mutation. *Current Opinion in Ophthalmology*.

[14] Vasyuta V. A. (2015). Study Effect of Assosiated Pathology on the Development of Optic Nerve Atrophy. *Lik Sprava*.

[15] Amorim S., Heise C. O., Santos S., Macedo-Souza L. I., Zatz M., Kok F. (2014). Nerve conduction studies in spastic paraplegia, optic atrophy, and neuropathy (SPOAN) syndrome. *Muscle & Nerve*.

[16] Singalavanija A., Hemarat K., Kedkovid N. (2013). Optic atrophy after anti-vascular endothelial growth factor injection in diabetic patients with proliferative diabetic retinopathy. *Journal of the Medical Association of Thailand*.

[17] Golnik K. (2010). Nonglaucomatous Optic Atrophy. *Neurologic Clinics*.

[18] Lechauve C., Augustin S., Cwerman-Thibault H. (2014). Neuroglobin gene therapy prevents optic atrophy and preserves durably visual function in harlequin mice. *Molecular Therapy*.

[19] Wei S. H., Zhang X. L. (2012). Comment on the therapeutic value of optic atrophy. *Zhonghua Yan Ke Za Zhi*.

[20] So K.-F., Aguayo A. J. (1985). Lengthy regrowth of cut axons from ganglion cells after peripheral nerve transplantation into the retina of adult rats. *Brain Research*.

[21] Heiduschka P., Thanos S. (2000). Restoration of the retinofugal pathway. *Progress in Retinal and Eye Research*.

[22] Schuetz E., Thanos S. (2004). Neuro-glial interactions in the adult rat retina after reaxotomy of ganglion cells: examination of neuron survival and phagocytic microglia using fluorescent tracers. *Brain Research Bulletin*.

[23] Newman N. J. (2012). Treatment of hereditary optic neuropathies. *Nature Reviews Neurology*.

[24] Chen C., Luo Y. (2016). Clinical study of acupuncture-moxibustion for optic atrophy. *Journal of Yunnan Chinese Medicine*.

[25] He H. L., Qian X. L., Shu S. (2011). Research progress of acupuncture-moxibustion in treating optic atrophy. *Journal of Yunnan Chinese Medicine*.

[26] Zhang H., Zhou J. F., Jin R. (1996). Effect of acupuncture on bulbar conjunctival microcirculation in optic atrophy patients. *Journal of Guangzhou University of Traditional Chinese Medicine*.

[27] Yuan X. J., Hao X. S., Lai Z. P., Zhao H., Liu W. Y. (1996). Effect of acupuncture at Feng-chi (GB20) on cerebral blood flow. *Journal of Traditional Chinese Medicine*.

[28] Wang F., Zhang K. Q. (2000). Anatomical observation of ophthalmic artery and branches. *Chinese Journal of Experimental Ophthalmology*.

[29] Zhu H. A., Li C. K., Peng Q. H. (2001). An experimental research about effect of acupuncturing tai-chong(LR3)acupoint on the P100 latent time of P-VEP. *China Journal of Chinese Ophthalmology*.

[30] Yu K. C., Fang B., Wang F. (2004). Efficacy observation of acupuncture plus medication for 11 cases of primary optic atrophy. *Journal of Heilongjiang Chinese Medicine*.

[31] Higgins J. P. T., Altman D. G., Higgins J. P. T., Green S., Sterne J. A. C. (2017). Assessing risk of bias in included studies. *Cochrane Handbook for Systematic Reviews of Interventions Version 5.1.0*.

[32] Liu C. Y., Qin S., Li Z. R. (2016). Observation on the efficacy of acupuncture at xin-ming points plus strong reinforcing manipulation in treating optic atrophy. *Shanghai Journal of Acupuncture and Moxibustion*.

[33] Zhao D. (2016). Clinical observation of acupuncture-moxibustion plus acupoint injection for optic atrophy. *Shaanxi Chinese Medicine*.

[34] Sun S. L. (2015). Efficacy observation of compound anisodine hydrobromide plus acupuncture for optic atrophy. *World Latest Medicine Information*.

[35] Wu X. M., Feng S. F., Zhou Z. A. (2010). Clinical study of compound anisodine hydrobromide plus acupuncture for optic atrophy. *Journal of Practical Traditional Chinese Medicine*.

[36] Xia Q. Y. (2010). Clinical observation of compound anisodine hydrobromide plus four-point eight-needle acupuncture for optic atrophy. *China Journal of Chinese Ophthalmology*.

[37] Dai H. B., Liu Y. A., Xia Y., Li H. (2009). Clinical characteristics and treatment analysis of optic atrophy. *Medical Information*.

[38] Tian X. G. (2018). Observation on Clinical Effects of Zheng's Warming Needling Method in Treating Optic Atrophy of Qi Stagnation and Blood Stasis Pattern. *Western Journal of Traditional Chinese Medicine*.

[39] Yin L. X., Duan L. (2012). Clinical observation of optic atrophy treated by local acupuncture. *Journal of Baotou Medical College*.

[40] Huang C. J. (2005). *Clinical research and basic research on acupuncture treatment of glaucomatous optic atrophy*.

[41] Law S. K., Li T. (2013). Acupuncture for glaucoma. *Cochrane Database of Systematic Reviews*.

[42] Lempert P. (2012). Acupuncture Therapy for Amblyopia. *Ophthalmology*.

[43] Kim T.-H., Kang J. W., Kim K. H. (2012). Acupuncture for the treatment of dry eye: a multicenter randomised controlled trial with active comparison intervention (artificial teardrops). *PLoS ONE*.

[44] Sabel B. A., Henrich-Noack P., Fedorov A., Gall C. (2011). Vision restoration after brain and retina damage: The "residual vision activation theory". *Progress in Brain Research*.

[45] Dai Y. L., Liu M., Zhang Y. X., Wei S. H., Huang H. B. (2013). Meta analysis of acupuncture in the treatment of optic atrophy. *Journal of Central South University. Medical Science*.

[46] Liu M. L., Lan L., Tang Y. (2009). An acupuncture meta-analysis for optic atrophy Seven randomized, controlled trials. *Neural Regeneration Research*.

[47] Schulz K. F., Altman D. G., Moher D. (2010). CONSORT 2010 statement: updated guidelines for reporting parallel group randomised trials. *BMC Medicine*.

[48] MacPherson H., Altman D. G., Hammerschlag R. (2010). Revised Standards for Reporting Interventions in Clinical Trials of Acupuncture (STRICTA): Extending the CONSORT Statement. *Plos Medicine*.

